# Wnt Sets the Stage for Spinal Cord Patterning in the Chick

**DOI:** 10.1371/journal.pbio.0040280

**Published:** 2006-07-18

**Authors:** Françoise Chanut

The spinal cord and brain harbor a variety of motor neurons that innervate limbs, thorax, neck, or face. Different classes of motor neurons reside at different locations along the head-to-tail (rostrocaudal) axis of the nervous system, reflecting a patterning that arises early in embryonic development, when the nervous system is but a simple tube. Indeed, different sections of the neural tube begin expressing distinct subsets of genes thought to govern cell fate, such as members of the *Hox* gene family, shortly after the tube forms. The question is how this patterned gene expression arises. Ulrika Nordström, Thomas Edlund, and their colleagues have tackled this problem in the chick embryo. They report that rostrocaudal patterning of the spinal cord and hindbrain is under the influence of the signaling molecule Wnt. Other signals refine Wnt patterning and create distinct sections in which different types of motor neurons eventually differentiate.

The embryonic nervous system of vertebrates is shaped by signals coming from surrounding tissues, in particular from the mesoderm, which gives rise to skeletal bones and muscles. The mesodermal signal fibroblast growth factor (FGF), for instance, imposes caudal fates on the developing nervous system, whereas Wnt and FGF in combination pattern the middle portions of the prospective brain. Since Wnt remains abundant in regions where the spinal cord will start to grow behind the nascent brain, Nordström and her colleagues reasoned that it might also pattern the posterior regions of the embryo’s nervous system.

The researchers cut out small fragments (called “explants”) of prospective neural tissue from chick embryos that had reached different developmental stages and observed the explants’ development in culture. At developmental stage 4, the chick embryo is a pin-head-size disc of cells that lies atop the yolk. Its most prominent feature is the primitive streak, a thin furrow that starts as an anterior dimple known as Hensen’s node and runs all the way to the embryo’s posterior end. Cells destined to become neural occupy a horseshoe-shaped halo around Hensen’s node. The anterior and middle portions of the brain derive from the horseshoe’s arch, whereas hindbrain and spinal cord come from its branches.

The researchers found that stage 4 explants taken from the horseshoe’s branch expressed *Hoxc9*, *Hoxb8* and *Hoxb4* in its caudal half and *Krox20* in its rostral half after a day in culture. The *Hox* gene combination is characteristic of the lumbar and tail region of the spinal cord, whereas expression of *Krox20* marks the anterior hindbrain. When cultured in the presence of an inhibitor of Wnt signaling, the explants failed to turn on *Krox20* and the *Hox* genes, and instead expressed *Otx2*, a marker of the forebrain. Therefore, Wnt signaling appears necessary for the neural tube to adopt the posterior characteristics of hindbrain and spinal cord.

Missing from the cultured explants, however, were cells expressing a combination of *Hoxb8* and *Hoxb4*, which mark the thoracic and neck regions of the spinal cord, or *Hoxb4* alone, which marks the posterior hindbrain. By contrast, these cells appeared readily in cultured explants taken from stage 8 embryos. By this stage, mesoderm has crept through the backward-sliding Hensen’s node to lie under the elongating neural tube, where it begins to organize into somites, the precursors of vertebrae and ribs. The researchers reasoned that retinoic acid, a signal secreted by somites, might combine with Wnt to specify hindbrain and anterior spinal cord fates.

Adding retinoic acid to cultures of stage 4 explants confirmed this hypothesis. retinoic acid suppressed the expression of *Krox20* and *Hoxc9*, while inducing the appearance of cells expressing *Hoxb8* and *Hoxb4*, or *Hoxb4* alone. If the cultures also contained FGF, which normally diffuses from the primitive streak, the explants turned mostly into *Hoxb8/b4*-expressing cells. Culturing stage 4 explants from the forebrain region in the presence of various combinations of Wnt with retinoic acid or FGF also led to the appearance of distinct rostrocaudal subsets of spinal cord markers.

The researchers propose that, during normal development, early Wnt signaling steers the growing neural tube toward posterior fates, while retinoic acid and FGF later subdivide it into smaller rostrocaudal domains from which distinct motor neurons emerge.

## 

**Figure:**
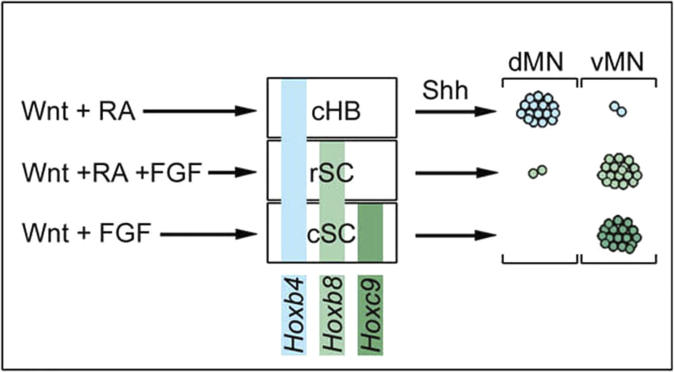
Combinatorial Wnt, retinoic acid, and FGF signals specify progenitor cell identity that prefigure motor neuron subtype in the developing hindbrain and spinal cord.

